# Intelligence, &c.

**Published:** 1826-04

**Authors:** 


### XVII.
### INTELLIGENCE, &c.

Victualling Office, 23rd February 1826.

The Right Honorable the Lords Commissioners of the Admiralty having been pleased to direct, "that no person be admitted to be a Candidate for the situation of Assistant Surgeon in the Royal Navy, who shall not produce a Certificate from one of the Royal Colleges of Surgeons of London, Edinburgh, and Dublin, of his fitness for that Office; nor for that of Surgeon, unless he shall produce a Diploma, or Certificate, from one of the said Royal Colleges, founded on an examination to be passed subsequently to his appointment of Assistant Surgeon, as to the Candidate's fitness for the situation of Surgeon in the Navy; and that in every case the Candidate producing such Certificate, or Diploma, shall also undergo a further examination before the Medical Commissioners of the Victualling Board, touching his qualifications in all the necessary branches and points of Medicine and Surgery for each of the steps in the Naval Medical Service;" The Commissioners for Victualling His Majesty's Navy, &c., do hereby signify, for the information of those persons to whom it may relate, that these regulations and directions will be strictly adhered to in future; and further, that previously to the admission of Assistant Surgeons into the Navy, it will be required that they should have received a classical education, and possess in particular a competent knowledge of Latin; also,

That they should have served an apprenticeship, or have been employed in an Apothecary's shop for not less than two years.

That their age should not be less than 20 years, nor more than 26 years.

That they should have attended an Hospital in London, Edinburgh, Dublin, or Glasgow, for 12 months; and

That they should have attended Lectures, &c. on the following subjects, for periods not less than hereunder stated, viz.

| | | |
|---|---|---|
| Anatomy ........ ................ | 18 | Months. |
| Surgery ..... ......... ........... | 18 | " |
| Theory of Medicine ......... ...... | 12 | " |
| Practice of ditto... ........ ... .... | 12 | " |
| Chemistry ................. .... | 6 | " |
| Materia Medica ............. .... | 6 | " |
| Midwifery ................ .... | 6 | " |
| Actual dissections of the human body.. | 6 | " |

Although the above are the only qualifications which are absolutely required in Candidates for the appointment of Assistant Surgeon, a preference will be given to those who, by possessing a knowledge of diseases of the Eye, and of any branch of science connected with the profession, such as Botany, Medical Jurisprudence, Natural Philosophy, &c. appear to be more peculiarly eligible for admission into the Service.

It is also to be observed that, by the rules of the Service, no Assistant Surgeon can be promoted to the rank of Surgeon until he shall have served full three years in the former capacity; and the Board have resolved that not any Diploma or Certificate of examination from either of the aforesaid Royal Colleges, shall be admitted towards the qualification for Surgeon, unless the Diploma or Certificate shall be obtained on an examination passed after a period of not less than three years service as Assistant Surgeon.

*By command of the Board,*

M. WALLER CLIFTON.

---

*An Obturateur of Gold, invented by Mr. J. Snell, Lecturer on the Teeth, &c.*

This instrument is so constructed that it may be adjusted in an aperture in the palatine portion of the maxillary bone, or palata mollis, with the greatest ease and facility by the wearer, being exceedingly simple in its application although complicated in its construction. It has the property of being retained in its proper situation without any attachment to the surrounding teeth, or without making the least pressure upon the sides of the cavity, being held up by wings covered with Indian rubber having a perpendicular movement, being depressed and elevated at pleasure by means of a key so constructed, that it shall be moved round by two cog wheels placed at right angles. The horizontal one being acted upon by a small ivory handle. This instrument is capable of being varied to every description of accidental perforation through the palate.

111, Crawford-street, Montague-square.

☞ Medical gentlemen interested in this subject, may have an opportunity of seeing the instrument, and the mode of its application, by calling upon Mr. J. Snell, at his address, between the hours of ten and four o'clock.

N.B. Dr. Johnson has seen this Obturateur, and considers it exceedingly ingenious in construction, and likely to answer every purpose for which it is designed.

---

MIDDLESEX INFIRMARY.

*No. 37, Great Pulteney Street, Golden Square.*

Medical officers. Dr. James Johnson, Dr. Joseph Ayre, Dr. Thomas Filkin, Mr. James Boyle, Mr. Thomas Alcock, Mr. George Jewel. A Physician and Surgeon attend every day, in rotation, *at one o'clock precisely.* Patients are received (for advice only) from Medical Practitioners of the Metropolis, on presenting a line from the gentleman under whose care the patient is. Other patients, with letters from subscribers, receive medicines as at other charitable institutions.

---

A medical gentleman, in genteel private practice, and most eligibly situated in the centre of the hospitals and medical schools, in the west end of the town, will take into his family one pupil, whose education he will superintend, while attending hospitals and lectures. The terms are moderate. Reference for the name and character of the gentleman may be made (by letter post paid) to Dr. James Johnson.

*To those whom it may concern.*

The letter of ATROPOS came to hand, full of friendly offers and insidious counsel. "*Timeo Danaos et dona ferentes.*" We suspect that it comes from the enemy's camp. At all events we want no assistance of the kind proffered; it is not with such weapons as ATROPOS would furnish, that we have hitherto maintained our ground—and we do not mean to change our mode of defence. We regard it indeed as a piece of peculiar good fortune to escape the praise, and daily receive the vituperation of the outcasts of medical society—of men, who have been accused of arson, convicted of libel, cast in a court of equity as literary robbers, and driven from the practice of a profession which they have disgraced—of men, who first attracted notice by obscenity, and can only maintain it by slander—of men, whose instincts prompt them to prey on garbage, and, as literary scavengers, to fling their dirt at every passenger—of men, whose courage consists in openly rifling the intellectual property of others, and abusing those whom they have robbed—of men, whose main talent consists in the art of *legerdemain* pilfering; and of pandering with most sagacity to the meanest passions—of men, whose liberality of sentiment is freedom from every scruple of conscience, or principle of rectitude—of men, who have never evinced, because they do not possess, one particle of professional knowledge beyond what they picked up in the Lecture-room—finally, of men, whose organized system of defamation will mark an æra, and fix a stain on the history of medicine, in the country which had the misfortune to give them birth!—Such are the men whose unremitting ire we have called forth, and whose ire, we trust in Heaven, will never remit, till malignity work (and work it will) the maligner's fall.

---

*Mr. Bell's Lectures at the College.*

Mr. Bell's first lecture at the College (the only one delivered before this sheet went to press) attracted great attention from a crowded audience. The principal subject was *counter-fissure* or fracture, and many ingenious arguments and illustrative cases were brought forward to shew the *principle* on which the counter-fissure takes place. The drawings were, of course, most beautiful—especially that of the sabre-wounded soldier. Mr. Bell concluded with some observations on phrenology, but he had not time for a clear development of his ideas on that intricate subject. The theatre was crowded to excess—but all went off quietly during and subsequent to the lecture. We understood that, previously to the admission of visitors, the appearance of a SALAMANDER in the gallery, among the students, excited considerable ferment there, and that, at one time, there was every reason to believe the fallen Angel's ambition would be gratified by an aerial flight *over the heads* of members, council, and all! The threatened expulsion of *Lucifer** from the presence of the gods, on high Olympus, spread terror and dismay among the inhabitants of the lower regions; but happily they were spared a visit from this *fire-brand,* or rather *fire-factor,* as he is now generally denominated, *who willingly,* for once, made his exit through the postern gate.

* The common derivation of this word is wrong. It is not from *Lux* and *fero*, but from *ignis* and *facio,* vide "*Secret* Memoirs of the *House* of *Argyll.*—Also "A New Mode of Lighting the Streets without Lamps," and "*Extinguishing Debts* by means of *Fire-Engines.*"

